# RMBNToolbox: random models for biochemical networks

**DOI:** 10.1186/1752-0509-1-22

**Published:** 2007-05-24

**Authors:** Tommi Aho, Olli-Pekka Smolander, Jari Niemi, Olli Yli-Harja

**Affiliations:** 1 Department of Information Technology, Institute of Signal Processing, Tampere University of Technology, Tampere, Finland; 2 Department of Information Technology, Institute of Mathematics, Tampere University of Technology, Tampere, Finland

## Abstract

**Background:**

There is an increasing interest to model biochemical and cell biological networks, as well as to the computational analysis of these models. The development of analysis methodologies and related software is rapid in the field. However, the number of available models is still relatively small and the model sizes remain limited. The lack of kinetic information is usually the limiting factor for the construction of detailed simulation models.

**Results:**

We present a computational toolbox for generating random biochemical network models which mimic real biochemical networks. The toolbox is called Random Models for Biochemical Networks. The toolbox works in the Matlab environment, and it makes it possible to generate various network structures, stoichiometries, kinetic laws for reactions, and parameters therein. The generation can be based on statistical rules and distributions, and more detailed information of real biochemical networks can be used in situations where it is known. The toolbox can be easily extended. The resulting network models can be exported in the format of Systems Biology Markup Language.

**Conclusion:**

While more information is accumulating on biochemical networks, random networks can be used as an intermediate step towards their better understanding. Random networks make it possible to study the effects of various network characteristics to the overall behavior of the network. Moreover, the construction of artificial network models provides the ground truth data needed in the validation of various computational methods in the fields of parameter estimation and data analysis.

## Background

Modeling and analysis of large biochemical networks is in its infancy. Networks' intrinsic capabilities and behavior arise both from the numerous network components and their complex interactions, thereby making the modeling task very challenging. In the field of computational systems biology, researchers modeling these networks often aim at predicting the system behavior in response to a given treatment. For example, lethality prediction for gene deletions [1,2] and maximization of the yield of a metabolic product [3,4] provide interesting applications.

Currently the structures of various biochemical networks are under extensive research. Best known are the structures of metabolic networks which are reconstructed on the basis of genome annotation, and biochemical and physiological evidence [5]. Metabolic network models are reconstructed e.g. for yeast *Saccharomyces cerevisiae *[1,6], bacteria *Escherichia coli *[7,8] and *Streptomyces coelicolor *[9], and a number of other organisms [10]. The structures of other intracellular networks types than metabolic networks are poorer known. Gene regulatory networks are explored in large scale in gene deletion studies [11-13] and transcription factor binding experiments [14], but various uncertainties relate to those studies. On the other hand, much information is available for protein-protein interaction networks and signal transduction networks [15-17] but, for example, the modular composition of proteins retards their reconstruction [18,19].

Besides structural information, the modeling of biochemical network behavior needs information about reaction kinetics, too. Reaction kinetics is much studied in biochemistry but, unfortunately, it still remains mostly unknown because of the difficult quantification of reaction velocities, especially *in vivo *[20,21]. In some cases it has been possible to construct kinetic models for reaction pathways [22]. In these situations, both the network structure and reaction kinetics are known, and the network model can be simulated using a system of ordinary differential equations (ODEs). However, in most cases the lack of kinetic information prevents the construction of ODE models or the model sizes remain very limited.

The usual approach to construct an ODE model for a biochemical pathway is to collect the needed information from literature piece by piece. The process is time consuming, and uncertainties appear in model construction because of natural complexity of cellular systems and the varying conditions in which they are examined.

A complementary method to construct ODE models is to adopt the available information, and then randomly generate the lacking information. These partially random models have several applications. First, they provide the ground truth data for objective evaluation of methods in data analysis and parameter estimation. The fundamental problem in those fields is that the goodness of the methods cannot be evaluated because data from real biological measurements is always noisy and the correct values remain unknown (see, e.g., [23-25]). Second, a researcher can generate a practically unlimited number of networks in which given features are varied. This makes it possible to study interrelationship between network structure and function, and to obtain statistical significance on the results (see, e.g., [26]). Third, the approach allows gradual model construction in which randomness is decreased after more information becomes available. For example, if the parameters of kinetic rate laws were previously drawn from a distribution, their values can be fixed when they become known. Thus, the model becomes more similar to its biological example.

There are many software for time series simulation, parameter estimation, and other analysis of biochemical network models (see, e.g. [27-34]). To authors' knowledge, however, there is no freely available an easily extendable software toolbox for generation of random ODE models for biochemical networks. The existing network generation softwares [35,36] have different modeling approaches and principles.

## Implementation

Research objectives may set various requirements for model generation. In the case of a metabolic network model generation, the network structure and its stoichiometry may be known, and only the kinetic laws have to be generated. In contrast, in generation of genetic regulatory network models, network structures are usually unknown, too, and their generation may be based on statistical rules. RMBNToolbox makes it possible to produce network models for various situations. However, there are infinitely different kinds of research objectives, and the toolbox may not be able to fulfill all the needs a user has. In order to help the user to implement her own functions easily, the toolbox has a modular structure, and the source code is freely available under GNU General Public Licence. RMBNToolbox is implemented in the Matlab environment [37] which provides a flexible environment for its further development. The toolbox is comprised of a set of Matlab functions which make the model construction possible when used together. It is illustrative to consider the network generation task using a compact mathematical framework. Especially for metabolic networks, the structural and kinetic information can be well summarized using a time variant concentration vector **c**, a time invariant stoichimetric matrix *S*, and a time variant reaction rate vector **v**. Vector **c **contains concentrations for all the *m *species (*c*_*i*_, *i *= 1, ... *m*). The *m *× *n *matrix *S *represents the network structure by storing stoichiometric coefficients of all *n *reactions in its columns. The element *S *(*i, j*) > 0 if reaction *j *produces species *i*, *S *(*i, j*) < 0 if reaction *j *consumes species *i*, and otherwise *S *(*i, j*) = 0. The reaction rate vector **v **describes reaction rates *v*_*j*_, *j *= 1, ... *n*. Reaction rates vary according to kinetic laws which are linear or nonlinear algebraic functions. Typically, kinetic laws determine the rates based on the amounts of species participating to reactions as well as various reaction specific parameters. Altogether, an ODE model can be formulated as

dcdt=Sv.
 MathType@MTEF@5@5@+=feaafiart1ev1aaatCvAUfKttLearuWrP9MDH5MBPbIqV92AaeXatLxBI9gBaebbnrfifHhDYfgasaacH8akY=wiFfYdH8Gipec8Eeeu0xXdbba9frFj0=OqFfea0dXdd9vqai=hGuQ8kuc9pgc9s8qqaq=dirpe0xb9q8qiLsFr0=vr0=vr0dc8meaabaqaciaacaGaaeqabaqabeGadaaakeaadaWcaaqaaiabdsgaKHqabiab=ngaJbqaaiabdsgaKjabdsha0baacqGH9aqpcqWGtbWucqWF2bGDieaacqGFUaGlaaa@36B3@.

The reaction rates *v*_*j*_, *j *= 1, ..., *n *are determined by kinetic laws *f*_*j *_as

*v*_*j *_= *f*_*j *_(**c**_*j*_, **p**_*j*_),

in which **c**_*j *_includes concentrations of species taking part in the reaction *j*, and **p**_*j *_contains the parameter values of the kinetic law.

In addition to the basic scheme shown in Eqs. 1 and 2, the model may contain other details such as assignment rules. An assignment rule makes it possible to assign a specific value for a variable independently from the system of differential equations above. The value may depend on time, species amounts, or whatever other model variables.

**Figure 1:**
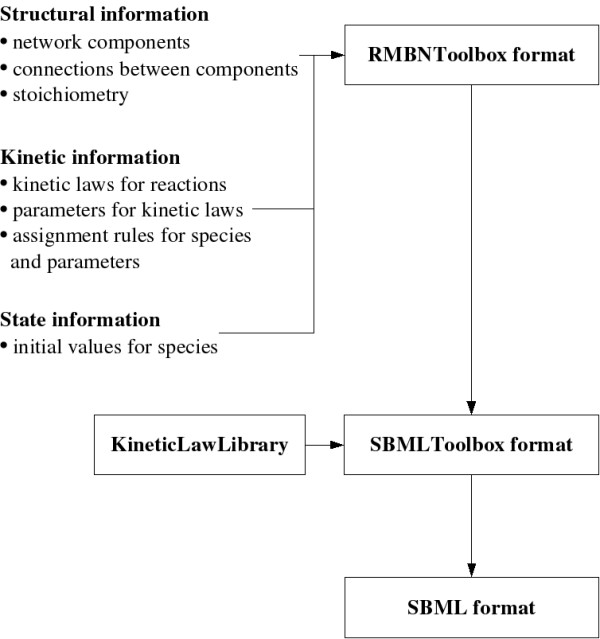
**Phases of model construction**. Structural, kinetic, and state information are prepared during model construction. Together with a library containing kinetic rate laws (KineticLawLibrary), the constructed model is converted to the format of SBMLToolbox. The model can be analyzed and exported in SBMLToolbox format, or it can be further converted to the format of Systems Biology Markup Language.

The generation of a biochemical network model using RMBNToolbox has three main steps. First, a network structure is determined. This includes defining both the network components, i.e., species and reactions, and their connections. As described in Section 'Network structure', the toolbox supports structure generation by providing a set of methods for random and deterministic approaches. In the second step of the network model generation, network kinetics are determined by setting kinetic laws for reactions, parameter values therein, possible assignment rules for species, etc. Section 'Network kinetics' describes how the toolbox makes it possible to accomplish these tasks. Before the network can be simulated, its initial state must be defined. Section 'Initial state of the network' takes a look at this task. Matlab scripts can be used to call the toolbox functions so that all the structural, kinetic, and state data are generated into the model. In practice, the model is constructed into a data structure which mainly exploits Matlab vectors, matrices, and their indexing. The details of the data structure and toolbox functions are described in the toolbox manual which is delivered along with the toolbox. The usage of toolbox functions is described in their help documentation. Figure 1 summarizes the main phases of model construction as well as further steps which are needed to export the generated model into the format of Systems Biology Markup Language (SBML).

### Network structure

The toolbox provides various methods for constructing network structures. The user can generate and import graph models as well as stoichiometric models. RMBNToolbox uses an incidence matrix representation to store a directed bipartite graph which describes the network structure. In the graph, species and reactions are nodes connected with directed edges. Edges indicate the direction of mass flow or controlling activity. Next we introduce the main approaches for setting up network structures.

The toolbox provides functions that make use of statistical rules in network structure generation. The user may specify the number of reactions, the number of species, and a probability density function. The probability density function defines the number of species that are connected to each reaction. For example, it may be required that the probabilities for reactions to have 1, 2 and 3 substrates, are 50%, 30%, and 20%, respectively. The method is useful when reactions have a known indegree distribution of substrates or outdegree distribution of products which is used as a determining feature for the structure generation. In various network systems, the structure of the network determines its stability. The toolbox offers a possibility to specifically generate stable or unstable linear systems as models for biochemical networks. Methods with different structure generation principles are implemented for this purpose. The first method generates network structures and tests their stability until a network structure with a stable (or unstable) behavior is found. The other methods generate network structures iteratively. One by one they connect random species to random reactions and check whether the network remains stable (or unstable). All the methods examine the model stability using the eigenvalues of the constructed system matrix. Further theoretical details are presented in an example of the Section 'Results'.

A network analysis study may be based on graph theoretical approaches, too. A tree is a graph in which no loops nor unconnected nodes exist. The toolbox makes it possible to generate trees as models of network structures. A tree sets up a network sceleton to which more reactions, species, or their connections can be added later on, or which can be analyzed further as such.

In addition to random structure generation, the user can specify any pre-defined network structure by providing a bipartite graph in the form of an *m *× *n *matrix *M*. In that case, the *m *rows represent species and *n *columns represent reactions, and the element *M *(*i, j*) equals one if species *i *is connected to reaction *j*. If a reaction and a species are not connected, then the respective element in *M *equals zero. This approach makes it possible for the user to easily generate any kind of network structure using her own methods, and to process the model further using the toolbox functions.

The toolbox supports the import of stoichiometric matrices. The user may find the import feature especially useful in the cases in which the structure of a metabolic network is known, but kinetics not. Stoichiometric matrices *S *are provided as *m *× *n *matrices (see, Equation 1), possibly along with the names for the *m *species and *n *reactions.

### Network kinetics

The main task in the generation of network kinetics is to choose and set kinetic laws for reactions in the network model (see, Eq. 2). RMBNToolbox has a function that randomly chooses kinetic laws from KineticLawLibrary [38] which contains many of the basic kinetic laws from biochemistry textbooks [20,21,39]. Kinetic laws have different forms depending on various features on their reaction mechanisms, such as the numbers of substrates and products, compulsory or arbitrary binding order of multiple substrates, and reversibility. Two features related to network structure determine if a specific kinetic law can be set for a specific reaction in the network model. First, the numbers of subsrates and products must be the same in the kinetic law and in the reaction it is applied to. Second, the reversibility of the kinetic law must match with the reversibility of the reaction. The choice of a kinetic law can be made randomly among those kinetic laws which fulfill these two requirements.

Kinetic laws have various parameters for which values need to be determined. By default, the parameter values are random numbers from uniform distributions. The user can redefine the distributions, and she can set new values separately for individual parameters if needed.

In addition to reactions, the amounts of species may be determined by assignment rules. In this case, the user writes an assignment rule as a Matlab M-file, and specifies the variables which are used for its evaluation. With a similar procedure, assignment rules can be set for parameters of kinetic rate laws. Thus, assignment rules make it possible for species and parameter values to be functions of any other variables.

### Initial state of the network

An initial state has to be given for a network model before its dynamical behavior can be simulated. This includes defining the initial amounts for species, but also the values of other time-dependent variables which may exist. The toolbox provides a function for this task. On the other hand, there are many network analysis methods that do not need the state information (e.g., flux balance analysis [7]). For those cases, the user can generate and export models without the state information.

### Exporting network models in SBML format

The network models created with the help of RMBNToolbox can be exported in the format of Systems Biology Markup Language (Level 2, version 1) [40]. An increasing amount of software tools support SBML for model exchange, and therefore the user can choose her favourite tool for further analysis of the generated models. RMBNToolbox bases its SBML support on other software. The network model generated by RMBNToolbox is converted to the format of SBMLToolbox [34] which is another toolbox working in Matlab. After kinetic laws are read from KineticLawLibrary [38] and added to the model, SBMLToolbox makes it possible to export the model in SBML format. The export is done with the help of the LibSBML library which is written in ISO C and C++ [41].

## Results

In this section we present examples of the intended use of RMBNToolbox. In the first example we generate a large model for a genetic regulatory network that can be used to produce ground truth data for a microarray simulation [25]. In the second example the structure and stoichiometry of a metabolic network are known, and the kinetic laws are randomly generated. Furthermore, the example demonstrates how metabolic fluxes in a steady state can be decomposed by elementary flux modes. The third example studies network stability using a control theoretic approach. The example generates small networks for which the network structure determines the stability. All the Matlab scripts that are used to generate the following example networks can be found in the examples folder of RMBNToolbox. All the generated example networks can be downloaded as additional files of this article.

### Gene regulatory network

In gene regulatory networks a set of genes produce proteins called transcriptional regulators. Transcriptional regulators bind to the promoter areas of genes, thereby activating or inhibiting their transcription. Most of the genes do not produce transcriptional regulators but their functions may be related to other processes, such as metabolism or cellular growth. Transcriptional regulators are usually thought as the key to the cellular control. In this example we produce a large network model with simple structural characteristics [see Additional file 1]. The model mimics a gene regulatory network.

In the generated network there are 1000 transcription reactions which produce one product each. The total of 200 of the products act as transcriptional regulators which control the network by activating and inhibiting the transcription reactions. Each of the transcription reactions has one activatory and one inhibitory regulator which are selected randomly from the 200 regulators.

The synthesis of the gene products is modeled similarly in all cases. The modeling concentrates on the kinetics of transcription and uses the rate law suggested in [23]. The amount of the protein product, for which the gene is a precursor, is assumed to be equal to the produced transcript. Because the number of gene copies is restricted and only a limited number of regulators are able to bind simultaneously, the kinetic law saturates both with the amount of activator and inhibitor. The rate of transcription is

r=VbasalKInIInI+KInIAnAAnA+KAnA,
 MathType@MTEF@5@5@+=feaafiart1ev1aaatCvAUfKttLearuWrP9MDH5MBPbIqV92AaeXatLxBI9gBaebbnrfifHhDYfgasaacH8akY=wiFfYdH8Gipec8Eeeu0xXdbba9frFj0=OqFfea0dXdd9vqai=hGuQ8kuc9pgc9s8qqaq=dirpe0xb9q8qiLsFr0=vr0=vr0dc8meaabaqaciaacaGaaeqabaqabeGadaaakeaacqWGYbGCcqGH9aqpcqWGwbGvdaWgaaWcbaGaemOyaiMaemyyaeMaem4CamNaemyyaeMaemiBaWgabeaakmaalaaabaGaem4saS0aa0baaSqaaiabdMeajbqaaiabd6gaUnaaBaaameaacqWGjbqsaeqaaaaaaOqaaiabdMeajnaaCaaaleqabaGaemOBa42aaSbaaWqaaiabdMeajbqabaaaaOGaey4kaSIaem4saS0aa0baaSqaaiabdMeajbqaaiabd6gaUnaaBaaameaacqWGjbqsaeqaaaaaaaGcdaWcaaqaaiabdgeabnaaCaaaleqabaGaemOBa42aaSbaaWqaaiabdgeabbqabaaaaaGcbaGaemyqae0aaWbaaSqabeaacqWGUbGBdaWgaaadbaGaemyqaeeabeaaaaGccqGHRaWkcqWGlbWsdaqhaaWcbaGaemyqaeeabaGaemOBa42aaSbaaWqaaiabdgeabbqabaaaaaaakiabcYcaSaaa@54F8@

where *V*_*basal *_is the rate of transcription in the absence of activators and inhibitors, *I *and *A *represent the concentrations of inhibitor and activator, *K*_*I *_and *K*_*A *_represent the concentrations with which the inhibitor and the activator have the effect of half of their maximal effects, and *n*_*I *_and *n*_*A *_act as Hill coeffcients. The parameter values are random numbers from the following uniform distributions: *V*_*basal *_∈ *U *(5,10), *K*_*I *_∈ *U *(2,3), *K*_*A *_∈ *U *(1,2), *n*_*I *_∈ *U *(1,2), *n*_*A *_*U *(1,2). The initial concentrations *I *and *A *are random numbers from the uniform distributions *I *∈ *U *(0, 1) and *A *∈ *U *(0, 1).

The degradation kinetics of each gene product follows the mass action law

*r *= *k P*,

where *k *is a rate parameter and *P *is the concentration of the gene product. Similarly to the kinetic laws of transcription reactions, the parameter values are unique for each degradation reaction. In this case, the value of parameter *k *is drawn from the uniform distribution *U *(0.01, 0.02).

For a comparison, we additionally simulate a duplicate network which mimics a gene deletion [see Additional file 2]. In the duplicated network, the production of a randomly chosen regulator is stopped by setting the parameter *V*_*basal *_of its transcription reaction to zero. Otherwise the duplicated network is identical to the original network.

**Figure 2:**
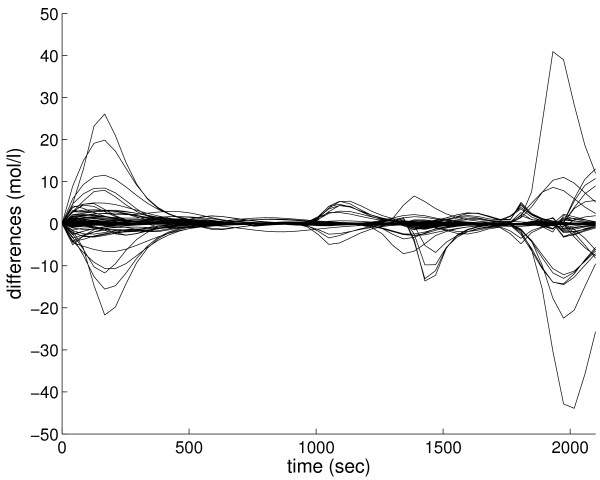
**Comparison of dynamical behavior**. The inactivation of a regulatory species in a gene regulatory network model affects dynamical behavior of the network. The differences in time series between the two simulations are plotted for each of the 1000 species.

The behavioral differences are illustrated by a time series simulation. After constructed, both networks are exported in SBML format and SBML ODE Solver [42] is used to simulate them. Figure 2 gives an overview to differences in simulation results. For each of the species, the figure shows concentration differences between the two simulations. For each time point *t*, the difference *d *(*t*) is calculated as

*d *(*t*) = *c *(*t*) - c* (*t*),

where *c *(*t*) and c* (*t*) are concentration values of the species in the first and in the second simulation, respectively. Although most of the species act similarly in both simulations, there are large and unforeseeable dynamic variations too. The effects of the inactivation of the regulator do not fade away or relax to a constant but the inactivation seems to have complex behavioral consequences.

### Simulation and stoichiometric analysis

In this example, a time series simulation is used to illustrate a result from stoichiometric network analysis. As presented in [43], any feasible steady state flux distribution of a metabolic network is a linear combination of so-called elementary flux modes (EFM). We show using random reaction kinetics that this holds in a small examplary metabolic network model. Extreme pathways [44] and elementary fluxes [45] are similar concepts to EFMs, and they would be equally valid for this analysis.

In metabolic systems, the time derivates of metabolite concentrations **c **can be written as presented in Equation 1, i.e., dcdt=Sv
 MathType@MTEF@5@5@+=feaafiart1ev1aaatCvAUfKttLearuWrP9MDH5MBPbIqV92AaeXatLxBI9gBaebbnrfifHhDYfgasaacH8akY=wiFfYdH8Gipec8Eeeu0xXdbba9frFj0=OqFfea0dXdd9vqai=hGuQ8kuc9pgc9s8qqaq=dirpe0xb9q8qiLsFr0=vr0=vr0dc8meaabaqaciaacaGaaeqabaqabeGadaaakeaadaWcaaqaaiabdsgaKHqabiab=ngaJbqaaiabdsgaKjabdsha0baacqGH9aqpcqWGtbWucqWF2bGDieaacqGFUaGlaaa@36B3@ where *S *is the *m *× *n *stoichiometric matrix with *m *metabolites and *n *reactions, and **v **contains the reaction velocites with *v*_*i *_≥ 0 for each irreversible reaction *i*. Metabolites are classified to external for which it is assumed that the environment always balances their concentrations **c**_*ext*_, and internal for which the concentrations **c**_*int *_are determined by the network. A network is then said to be in a steady state if

dcintdt=0,
 MathType@MTEF@5@5@+=feaafiart1ev1aaatCvAUfKttLearuWrP9MDH5MBPbIqV92AaeXatLxBI9gBaebbnrfifHhDYfgasaacH8akY=wiFfYdH8Gipec8Eeeu0xXdbba9frFj0=OqFfea0dXdd9vqai=hGuQ8kuc9pgc9s8qqaq=dirpe0xb9q8qiLsFr0=vr0=vr0dc8meaabaqaciaacaGaaeqabaqabeGadaaakeaadaWcaaqaaiabdsgaKHqabiab=ngaJnaaBaaaleaaieGacqGFPbqAcqGFUbGBcqGF0baDaeqaaaGcbaGaemizaqMaemiDaqhaaiabg2da9iab=bdaWiabcYcaSaaa@3952@

i.e., there is no accumulation or depletion of internal metabolites. Specific reaction velocities (flux distributions) are needed to maintain steady states.

Elementary flux modes describe such reversible and irreversible pathways in the network which maintain steady states when working. In an elementary flux mode, each reaction is assigned with its relative velocity compared to other reactions in the same EFM. EFMs are minimal in the sense that the active reactions in an EFM cannot be a subset of the active reactions in another EFM. Elementary flux modes can be calculated based on a stoichiometric matrix and the respective reaction irreversibilities [43]. Let vector **e **denote an elementary flux mode in which element *e*_*i *_= 0 if reaction *i *is inactive and *e*_*i *_≠ 0 if the reaction *i *is active. Further, let the set of all *N *elementary flux modes of the network be in matrix *E *= [**e**_1_, **e**_2_, ..., **e**_*N*_]. Then any flux distribution **v**, which results a steady state into the network, can be described as a linear combination of the EFMs as

**v **= *E β*,   *β*_*j *_≥ 0 if EFM *j *is irreversible

where the vector *β *weigths each of the elementary fluxes by a scalar. The weigths are non-negative for EFMs describing irreversible pathways.

Because the calculation of EFMs uses the steady state assumption, Equation 7 has solutions for *β *only if **v **maintains a steady state. Usually the number of EFMs (columns in *E*) is much larger than the number of reactions (rows in *E*), and therefore unique solutions are rare for Equation 7. However, we can test the existence of solutions by setting up a linear programming problem

max⁡1Tβsuch thatEβ=vβj≥0,EFM jisirreversible
 MathType@MTEF@5@5@+=feaafiart1ev1aaatCvAUfKttLearuWrP9MDH5MBPbIqV92AaeXatLxBI9gBaebbnrfifHhDYfgasaacH8akY=wiFfYdH8Gipec8Eeeu0xXdbba9frFj0=OqFfea0dXdd9vqai=hGuQ8kuc9pgc9s8qqaq=dirpe0xb9q8qiLsFr0=vr0=vr0dc8meaabaqaciaacaGaaeqabaqabeGadaaakqaabeqaaiGbc2gaTjabcggaHjabcIha4Hqabiab=fdaXmaaCaaaleqabaacbaGae4hvaqfaaGGacOGae0NSdigabaqbaeaabiGaaaqaaiab+nhaZjab+vha1jab+ngaJjab+HgaOjabbccaGiab+rha0jab+HgaOjab+fgaHjab+rha0bqaaiabdweafjab9j7aIHGaaiab81da9iab=zha2bqaaaqaaiab9j7aInaaBaaaleaacqWGQbGAaeqaaOGaeyyzImRaeGimaaJaeiilaWIae4xrauKae4NrayKae4xta0KaeeiiaaccbiGaeSNAaOMae4hiaaIae4xAaKMae43CamNae4hiaaIae4xAaKMae4NCaiNae4NCaiNae4xzauMae4NDayNae4xzauMae4NCaiNae43CamNae4xAaKMae4NyaiMae4hBaWMae4xzaugaaaaaaa@6676@

The objective function is set to find the maximum of the sum of the weigths. Rather than the maximum value, we are now interested in the existence of any solution. In the following, we utilize the fact that the maximum can be found only if any solutions exist.

**Figure 3:**
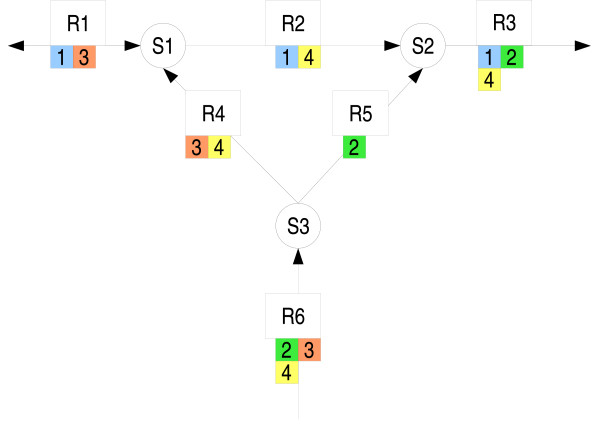
**Metabolic network and elementary flux modes**. Species are represented as circles and reactions as rectangles. Reaction stoichiometries are ones. The network has four elementary flux modes that are illustrated using small numbered squares with different colors. For example, mode 3 uses reactions R6, R4, and R1.

A hypothetical example network, illustrated in Figure 3, consists of three species and six reactions [46]. The network structure is provided to RMBNToolbox as a stoichiometric matrix. Initial amounts for metabolites, kinetic rate laws for reactions, and their parameter values are chosen randomly, because we aim at illustrating that an arbitrary steady state flux distribution is a linear combination of elementary flux modes.

**Figure 4:**
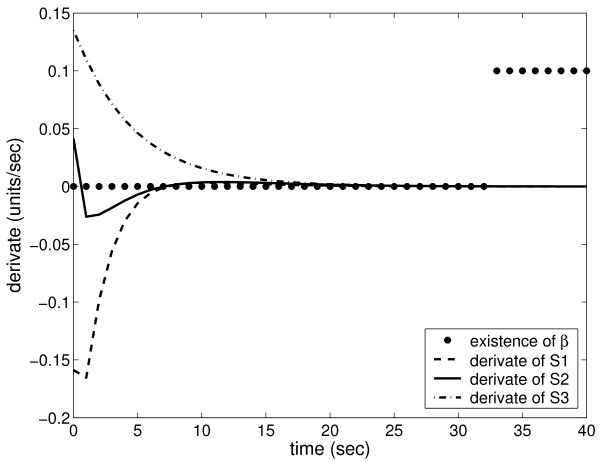
**Steady state requirement for elementary flux modes**. In a simulation the metabolic network gradually relaxes to a steady state in which the time derivates of internal species S1, S2, and S3 are zero. The existence of weights *β *is calculated for flux distributions sampled from the simulation time series. For illustration purposes, existence is coded with values 0 and 0.1 for nonexistence and existence, respectively. The weights *β *exist (i.e. a flux distribution can be decomposed using elementary flux modes) after a steady state is obtained.

Program Metatool [47] is used to calculate the elementary flux modes of the generated network. The network is exported in SBML format [see Additional file 3] and simulated using SBML ODE Solver [42] until a steady state is reached. During the simulation, the flux distribution **v **and time derivates of species amounts dcdt
 MathType@MTEF@5@5@+=feaafiart1ev1aaatCvAUfKttLearuWrP9MDH5MBPbIqV92AaeXatLxBI9gBaebbnrfifHhDYfgasaacH8akY=wiFfYdH8Gipec8Eeeu0xXdbba9frFj0=OqFfea0dXdd9vqai=hGuQ8kuc9pgc9s8qqaq=dirpe0xb9q8qiLsFr0=vr0=vr0dc8meaabaqaciaacaGaaeqabaqabeGadaaakeaadaWcaaqaaiabdsgaKHqabiab=ngaJbqaaiabdsgaKjabdsha0baaaaa@3224@ are sampled for every second. Linear programming problems, as described in Eq. 8, are solved for each flux distribution sample. Figure 4 shows the time derivates and the existence of weights *β *for each sample. After a steady state is reached (i.e., the time derivates of internal metabolites become zero), then the flux distribution is a linear combination of the elementary flux modes (i.e., the weigths *β *are found).

### Network stability

Neither RMBNToolbox nor Systems Biology Markup Language take care of the rationality of the generated network models. Possible unstability of the generated model is a typical issue the user has to consider. In this example we look how to exploit control theory for generating models which are unstable and biologically unreasonable and, on the other hand, stable and biologically more reasonable.

In control theory, a system is stable if it has a bounded response to a bounded input [48]. We concentrate on network models which can be formulated as systems of first-order linear differential equations. Their compact representation form is

dcdt=Ac,
 MathType@MTEF@5@5@+=feaafiart1ev1aaatCvAUfKttLearuWrP9MDH5MBPbIqV92AaeXatLxBI9gBaebbnrfifHhDYfgasaacH8akY=wiFfYdH8Gipec8Eeeu0xXdbba9frFj0=OqFfea0dXdd9vqai=hGuQ8kuc9pgc9s8qqaq=dirpe0xb9q8qiLsFr0=vr0=vr0dc8meaabaqaciaacaGaaeqabaqabeGadaaakeaadaWcaaqaaiabdsgaKHqabiab=ngaJbqaaiabdsgaKjabdsha0baacqGH9aqpcqWGbbqqcqWFJbWycqWFSaalaaa@365D@

in which the vector **c **contains states of variables, and the system matrix *A *determines the system properties. The system is known to be stable if the eigenvalues of *A *have nonpositive real parts, and every eigenvalue with zero as the real part has an associated Jordan block of order one [49,50].

Next we derive a model for which the user can determine if this stability requirement is fulfilled or not. For this purpose, the biochemical network model described by Equations 1 and 2 needs to be represented in the format of Equation 9.

We begin the model reformulation from kinetic laws, i.e., Equation 2. Because the intended model in Equation 9 is linear, we can utilize for its construction such kinetic laws which are linear too. Kinetic laws of the form first-order mass-action fulfill this need. For example, the kinetic law for reaction *j *is *v*_*j *_= *k*_*j *_*c*_*j *_where *k*_*j *_is a reaction-specific rate constant and *c*_*j *_is the concentration of the subsrate. Kinetic laws of this form make it possible to represent the reaction rate vector **v **of Equation 1 by a matrix-vector multiplication

**v **= Γ**c**,

where Γ is a diagonal *n *× *m *matrix storing rate constants *k*_*j*_, for each reaction *j *= 1, ..., *n*, on its main diagonal.

Substituting this to Equation 1, it becomes

dcdt=Sv=SΓc.
 MathType@MTEF@5@5@+=feaafiart1ev1aaatCvAUfKttLearuWrP9MDH5MBPbIqV92AaeXatLxBI9gBaebbnrfifHhDYfgasaacH8akY=wiFfYdH8Gipec8Eeeu0xXdbba9frFj0=OqFfea0dXdd9vqai=hGuQ8kuc9pgc9s8qqaq=dirpe0xb9q8qiLsFr0=vr0=vr0dc8meaabaqaciaacaGaaeqabaqabeGadaaakeaadaWcaaqaaiabdsgaKHqabiab=ngaJbqaaiabdsgaKjabdsha0baacqGH9aqpcqWGtbWucqWF2bGDcqGH9aqpcqWGtbWucqqHtoWrcqWFJbWycqGGUaGlaaa@3B96@

Multiplication of the time invariant matrices *S *and Γ results to the *m *× *m *matrix *A*. The substition of *A *to Equation 11 brings the network model to the form presented in Equation 9.

During the model construction, matrices *S *and Γ are randomly generated after which they are multiplied to produce the matrix *A*. The eigenvalues of *A *are calculated, and the values of their real parts are examined. The generation is repeated until the eigenvalues indicate the required stable or unstable behavior of the model.

**Figure 5:**
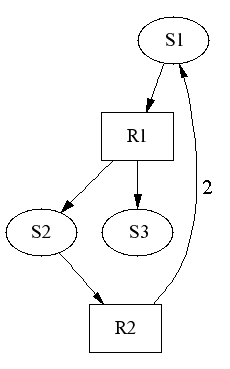
**Unstable model**. An unstable model has an unrealistic feedback loop that decomposes species S1 without loss of mass. Reaction stoichiometries are 1, except for reaction R2 that produces two molecules of S1.

**Figure 6:**
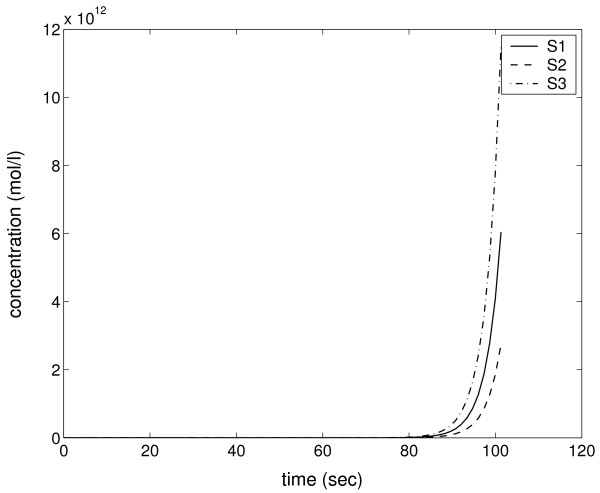
**Time series of unstable model**. Species amounts increase rapidly towards infinity in the simulation of the unstable model.

As the first example, we generate an unstable network model [see Additional file 4] by requiring at least one positive eigenvalue for the system matrix *A*. The structure of the generated network is depicted in Figure 5. We note that the network includes two kinds of features that are not reasonable in real biochemical networks and which obviously cause instability for the model. The first unrealistic feature is that the mass balance does not hold: Species S1 is decomposed in two parts, S2 and S3, in reaction R1. Further, reaction R2 converts one S2 molecule back to two S1 molecules. This results that S1 can be decomposed to S2 and S3 without loss of mass and, after each decomposition, the amount of S1 becomes doubled. The second problematic feature is the generation of dead-ends in the model: Species S3 is produced by reaction R1, but it is not consumed by any reaction. Therefore, the amount of S3 increases as long as there is a supply of S1. All this causes unstable behavior for the model, as demonstrated by Figure 6 in which species amounts increase rapidly towards infinity.

**Figure 7:**
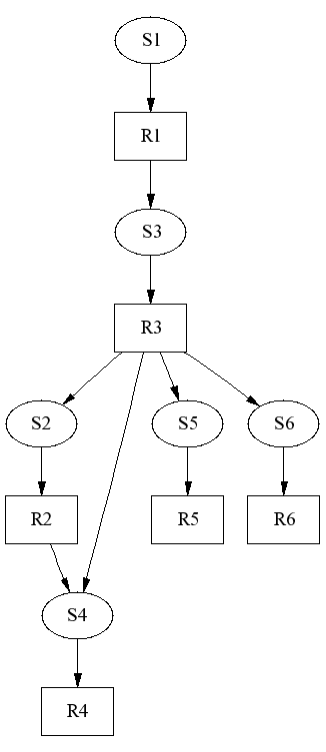
**Stable model**. A stable model does not have feedback loops that decompose and reuse species similarly to the unstable model. In contrast, the generated network structure is unidirectional. Reaction stoichiometries are ones.

**Figure 8:**
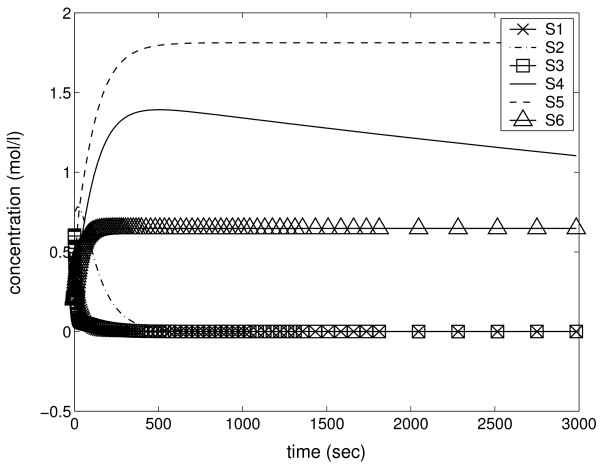
**Time series of stable model**. Species concentrations decrease to zero in the simulation of the stable model. This is because mass flows out of the network through reactions R4, R5, and R6. Species S4 remains longest in the network. Its concentration decreases below 0.05 mol/l after 2.91 * 10^5 ^seconds (data not shown).

In the second example the generated network model [Additional file 5] is stable. We note from Figure 7 that the network does not have the two structural features which appeared in the previous model. Instead, the structure is more treelike and without feedback loops, thereby enabling material flows through the network. Correspondingly, the species amounts show relaxation towards constant values in Figure 8.

## Discussion

In network model generation, the user has to define various network characteristics that include network components, their connections, stoichiometries, kinetic rate laws, etc. RMBNToolbox helps the user in these tasks by providing functions that make it possible to create and modify various structural and kinetic features. Many generation tasks are automated and, on the other hand, randomization can be exploited efficiently. The most of the features specified in Systems Biology Markup Language are supported by the toolbox. The toolbox does not oversee the rationality of the generated models, because an unreasonable model in one context may be reasonable in another one.

The model generation times are fair even for large models. For example, the genetic regulatory network model presented in Section 'Results' has 1,000 species and 2,000 reactions. The model and the corresponding SBML file were generated in appr. 40 seconds using a PC with 1GB RAM and Pentium M 1,3 GHz processor. Small network models, such as the one used in the stoichiometric analysis example, are generated within one second.

## Conclusion

We have presented a software called RMBNToolbox that can be used to generate random models for biochemical networks. The toolbox functions make it possible to generate network models with various user specified characteristics. For example, network structure, stoichiometric coefficients, kinetic laws and parameter values can be easily generated and manipulated. With the help of SBMLToolbox and LibSBML, the models can be translated into the format of Systems Biology Markup Language. The generated network models can be simulated and analyzed using any software that is able to use models provided in SBML format. The toolbox can be easily extended and modified, because it has a modular structure, it is implemented in Matlab environment, and it is freely available under GNU General Public Licence. Random network models can be applied to various purposes in the field of biochemical network modeling. Artificial models are needed to produce noise free data in which the characteristics are precisely known. Only that kind of data can be used for objective evaluation of various data analysis and parameter estimation methods. On the other hand, the new information acquired from biochemical networks can be included into the network model generation. This makes it possible to refine the model gradually while preserving the variations of the unknown parts of the network. Further, it is possible to study various emergent properties in network behavior, such as the effects of varying network connectivity. For these kinds of purposes, a sufficiently large number of network models is generated and the features of interest are varied.

## Availability and requirements

Project name: RMBNToolbox

Project home page: 

Operating system(s): Platform independent

Programming language: Matlab

Other requirements: LibSBML 2.3.2 or higher, SBMLToolbox 2.0.0 or higher

License: GNU GPL

## Authors' contributions

TA designed and implemented the main data structures and functions of the toolbox. O-PS and JN designed and implemented the functions related to control theory. OY-H initiated the study and participated to the coordination. All authors read and approved the final manuscript.

## Supplementary Material

Additional file 1 **Model for gene regulatory network**. Gene regulatory network model presented in the first example (in SBML format). Click here for file

Additional file 2 **Model for gene regulatory network with a gene deletion**. Modified gene regulatory network model presented in the first example (in SBML format). Click here for file

Additional file 3 **Model for metabolic network**. Metabolic network model presented in the second example (in SBML format). Click here for file

Additional file 4 **Model for unstable linear system**. Unstable linear system model presented in the third example (in SBML format). Click here for file

Additional file 5 **Model for stable linear system**. Stable linear system model presented in the third example (in SBML format). Click here for file
